# Integrated Omics Analysis of the Effects of Nano-Antimicrobial Peptide on the Intestinal Microbiota and Metabolome of Tibetan Sheep

**DOI:** 10.3390/ani16101543

**Published:** 2026-05-18

**Authors:** Yaqin Zhao, Xiaoshan Wang, Haixia Jing, Liyuan Zhao, Fengjun Liu

**Affiliations:** 1 Department of Veterinary Medicine, College of Agriculture and Animal Husbandry, Qinghai University, Xining 810016, China; yaqinzhao0809@163.com (Y.Z.); wangxiaoshan1202@163.com (X.W.); jinghaixia1@163.com (H.J.); 2 Qinghai Vocational and Technical Institute of Animal Husbandry and Vet, Xining 812100, China; qmzyuan@163.com

**Keywords:** Tibetan sheep, nano-antimicrobial peptide, 16S rRNA gene, non-targeted metabolomics

## Abstract

This study investigated the effects of dietary nano-antimicrobial peptides (NAP) on the microbial communities and metabolic profiles in Tibetan sheep. Using 16S rRNA gene high-throughput sequencing and non-targeted metabolomics, the contents of the small intestine, rumen, and rectum were systematically analyzed in a control group (Group A) and a NAP-supplemented group (Group B). Multi-omics integration methods, including O2PLS and Pearson correlation analysis, were employed to explore the association between microbial communities and metabolites. Alpha and beta diversity analyses revealed significant differences (*p* < 0.05) in the microbial community structure of the small intestine between the two groups. In contrast, the rumen and rectal microbiota remained relatively stable, indicating that the regulatory effects of NAP on the intestinal microecology are site-specific. In the small intestine, NAP altered the composition of dominant functional microbiota and the abundance of taxa related to energy metabolism. Metabolomic analysis identified significant shifts in metabolic profiles, specifically within the bile acid, fatty acid, and phospholipid pathways (*p* < 0.05). Group A exhibited baseline steady-state characteristics (e.g., cholic acids and phospholipids), whereas Group B showed activation of unsaturated fatty acids and related metabolites. Multi-omics integration revealed a stable systematic association between intestinal microbial genera and metabolites. Specifically, bile acid and prostaglandin metabolites were negatively correlated with *Firmicutes*-related taxa. These findings suggest that NAP supplementation may contribute to maintaining host energy metabolism and intestinal homeostasis by regulating intestinal microecology.

## Simple Summary

Nano-antimicrobial peptides are considered promising alternatives to conventional antibiotics. This study found that dietary supplementation with nano-antimicrobial peptides significantly affected the small intestinal microbiota of Tibetan sheep and altered the levels of conjugated bile acids, intermediate metabolites involved in phospholipid metabolism, and unsaturated fatty acids. These results suggest that nano-antimicrobial peptides may serve as potential alternatives to antibiotics in Tibetan sheep.

## 1. Introduction

Tibetan sheep (*Ovis aries*), a unique and valuable livestock resource on the Qinghai–Tibet Plateau, are primarily distributed in the high-altitude pastoral regions of Qinghai, Tibet, and Gansu provinces, at elevations of around 4000 m above sea level. They play a vital role in plateau animal husbandry and the local economy [1]. Over long periods of natural selection, Tibetan sheep have developed a specialized intestinal microecological system that enables them to adapt to harsh environments, including extreme cold, hypoxia, and limited forage availability [2]. The energy acquisition, nutrient utilization, and immune homeostasis of Tibetan sheep depend heavily on a stable and functionally efficient intestinal microbiota. Compared with common ruminants, the rumen microbiota of Tibetan sheep is characterized by a higher abundance of fiber-degrading taxa at the compositional level, as well as by functional features associated with adaptation to alpine environments, including the upregulation of glycosyltransferases (GTs) and carbohydrate-binding modules (CBMs). In addition, metabolites such as propionic acid, butyric acid, and lipid-related compounds may help meet energy demands, while a unique vascular structure and anti-inflammatory mechanisms contribute to the maintenance of physiological homeostasis. Together, these features may underlie some of the key physiological differences between Tibetan sheep and common ruminants [3,4].

The intestinal microbiota is a critical regulator of host health, influencing nutrient metabolism, immune function, and disease prevention [5,6,7]. Previous studies have shown that the microbiota of Tibetan sheep is primarily composed of dominant phyla such as *Firmicutes*, *Bacteroidetes*, *Proteobacteria*, and *Actinobacteria* [8,9]. These microorganisms play essential roles in energy metabolism, immune regulation, and the environmental adaptation of plateau ruminants [10,11,12,13]. Despite the importance of this microbial community, traditional breeding practices continue to rely heavily on antibiotics to regulate gastrointestinal health. Long-term use of antibiotics, however, can lead to bacterial resistance and food safety concerns, which challenges the sustainable development of animal husbandry [14].

Antimicrobial peptides (AMPs), which are natural host defense molecules, exhibit broad-spectrum antibacterial activity and have a lower risk of inducing antimicrobial resistance, making them promising alternatives to antibiotics [15]. Paneth cells, a key source of AMPs, play a crucial role in regulating the intestinal microbiota and maintaining the mucosal barrier by secreting antimicrobial peptides such as α-defensins and lysozyme [7]. AMP supplementation has been shown to enhance piglet weight gain, improve dairy cow milk quality, and optimize ewe lactation performance [15,16,17]. However, the application of natural AMPs is limited by several factors, including their susceptibility to proteolytic degradation in the gastrointestinal tract and their reduced bioavailability resulting from rapid renal and hepatic clearance. In addition, natural AMPs may exhibit cytotoxicity at high concentrations [18]. Recent advancements in nanotechnology have further enhanced the stability and antibacterial efficacy of AMPs, making them more suitable for use in complex physiological environments and extending their in vivo half-life [19,20,21]. Nanocarrier-based delivery systems can help improve the bioavailability and biocompatibility of antimicrobial peptides, reduce cytotoxicity, and achieve precise and targeted delivery against Gram-negative pathogens [22]. Previous studies have shown that nano-antimicrobial peptide (NAP) can not only promote intestinal development in lambs but also improve growth performance and meat quality [23]. Meanwhile, composite nano-antimicrobial peptides exhibit beneficial preventive and therapeutic effects on mastitis in dairy goats [24]. However, current studies on ruminants have mainly focused on growth performance and immune function. In particular, research related to Tibetan sheep remains scarce, and the mechanism underlying their intestinal microecological response to nano-antimicrobial peptides is still unclear.

To address this gap, this study aimed to evaluate the effects of dietary NAP on the intestinal microbiota of Tibetan sheep, using 16S rRNA gene sequencing and non-targeted metabolomics. The goal was to provide a theoretical foundation for green breeding of Tibetan sheep in alpine pastoral areas and the development of novel antibiotic-free feed additives. In this study, the rumen, small intestine, and rectum were selected as sampling sites. The small intestine is the primary site for nutrient digestion and absorption, and its mucosal structure and microbiota directly influence digestive efficiency and host health. As a specialized digestive organ in ruminants, the rumen plays a central role in feed degradation, volatile fatty acid production, and microbial metabolism. The rectum, located at the distal end of the gastrointestinal tract, serves as an indirect indicator of the microecological status of the distal gut. Together, these three sites represent distinct functional regions of the gastrointestinal tract in Tibetan sheep, allowing for a comprehensive understanding of the effects and mechanisms of nano-antimicrobial peptides in the gastrointestinal tract.

## 2. Materials and Methods

### 2.1. Ethics Approval

All experimental procedures were approved by the Institutional Animal Care and Use Committee (IACUC) of the College of Veterinary Medicine, Qinghai University (Approval No: SL-2023042; Approval Date: 29 July 2023). Every effort was made to minimize animal suffering and to reduce the number of animals used.

### 2.2. Animals and Experimental Design

Eighteen non-pregnant Tibetan sheep (35 ± 3 kg; 2–3 years of age) were obtained from the experimental station of Qinghai University, Qinghai, China. The sheep were randomly assigned to two groups (n = 9 per group): Group A (Control group), which was fed a basal diet consisting of 500 g of concentrate and 300 g of hay per sheep per day (note [25,26]); and Group B (Experimental group), which was fed the same basal diet supplemented with 0.125 g of nano-antimicrobial peptides (NAP) per sheep per day (note [27]).

The NAP was produced by recombinant expression technology through the construction of an expression vector containing linked Cecropin A and D sequences, followed by transformation into eukaryotic cells, large-scale culture, extraction, and purification. The peptide was subsequently encapsulated into polymer nanoparticles using an emulsification method. The nanoparticles exhibited a particle size distribution of 100–200 nm, a zeta potential of −20 mV, an irregular spherical morphology, and an encapsulation efficiency of 87.5%. The NAP used in this study was Cecropin AD, provided by Shaanxi Weiling Non-resistant Culture Technology Co., Ltd. (Shaanxi, China) (Q/DXN063-2021). Its amino acid sequence was as follows:

RSQKKVRFLLFVLTNKWAVVYNKCVEVDASSQTKRGNYDSETETGQSFRFVVMCTLFLSLPITETVL

The formulated feed had the following guaranteed composition: moisture ≤ 12.5%, crude protein ≥ 16.0%, crude fiber ≤ 9.0%, lysine ≥ 0.6%, calcium 0.7–1.2%, total phosphorus ≥ 0.6%, and sodium chloride 0.8–1.2%. The primary ingredients of the formulated feed included corn, wheat, bran, soybean meal, vegetable meal, cottonseed meal, calcium carbonate, sodium chloride, vitamins, trace elements, and NSP enzymes.

The hay consisted of oats and contained 9.8% crude protein, 58.3% neutral detergent fiber, 34.1% acid detergent fiber, and 2.1% crude fat.

All experimental sheep were maintained under consistent housing conditions with adequate ventilation and stable lighting. Before the formal experiment, all animals underwent a 14-day adaptation period to gradually acclimate to the experimental environment, basal diet, and daily feeding procedures, thereby minimizing environmental and dietary stress. During the experimental period, sheep were fed twice daily at fixed times (08:00 and 18:00), with concentrate and hay provided separately. The health status of the animals was monitored daily by a veterinarian throughout the 30-day experimental period.

### 2.3. Sample Collection

After 30 days of feeding, all sheep were slaughtered for sample collection. The animals were anesthetized via intravenous injection of sodium pentobarbital (20 mg/kg) and then exsanguinated without prior fasting in order to preserve the natural gastrointestinal contents.

Following laparotomy, the small intestine, rumen, and rectum were isolated. Contents from these three sites were collected and systematically labeled. For Group A, samples were designated as A-a (small intestine), A-b (rumen), and A-c (rectum); Group B samples were similarly labeled as B-a, B-b, and B-c. Each sample (approx. 2 mL) was immediately transferred into sterile 2.5 mL cryovials, snap-frozen in liquid nitrogen, and subsequently transported on dry ice to Gene Denovo Biotechnology Co., Ltd. (Guangzhou, China) for 16S rRNA gene sequencing and metabolomic analysis.

### 2.4. Bacterial 16S rRNA Gene Sequencing and Analysis

Total genomic DNA was extracted from the gastrointestinal contents using the HiPure Stool DNA Kit (Meiji Biotechnology Co., Ltd., Guangzhou, China) according to the manufacturer’s instructions. The concentration and purity of the extracted DNA were assessed using a NanoDrop 2000 spectrophotometer (Thermo Fisher Scientific, Waltham, MA, USA) to ensure sufficient quality for downstream applications. The V3–V4 hypervariable region of the bacterial 16S rRNA gene was amplified using the universal primers 341F (5′-CCTACGGGNGGCWGCAG-3′) and 806R (5′-GACTACHVGGGTATCTAATCC-3′). PCR amplification was performed using a high-fidelity enzyme system in triplicate to ensure technical consistency. The resulting amplicons were purified using AMPure XP Beads (Beckman Coulter, Brea, CA, USA) and quantified with a Qubit 3.0 Fluorometer (Thermo Fisher Scientific, Waltham, MA, USA). Sequencing libraries were constructed using the Nextera XT Index Kit (Illumina, San Diego, CA, USA) with unique sample barcodes. After library quality validation, 2 × 300 bp paired-end sequencing was performed on the Illumina MiSeq platform. Raw paired-end reads were first filtered to remove low-quality sequences and then assembled into tags. These tags underwent further quality control to generate clean tags. Subsequently, sequences were clustered into OTUs, during which chimeric sequences were identified and removed to yield effective tags. Taxonomic classification was assigned to the representative sequence of each OTU using the SILVA database (Release 138) with a confidence threshold of 0.8. Based on the OTU abundance profile, subsequent analyses, including alpha and beta diversity, as well as functional prediction, were performed. This study primarily focused on changes in microbial composition and community diversity at the phylum and genus levels, rather than on strain-level resolution. Therefore, OTU clustering was considered sufficient for the objectives of the present analysis. However, compared with the ASV-based approach, OTU clustering provides lower taxonomic resolution and may not effectively distinguish subtle sequence variations, which represents a methodological limitation of this study.

### 2.5. LC-MS Non-Targeted Metabolomics Detection and Analysis

Samples were thawed and extracted using a pre-cooled mixture of methanol, acetonitrile, and water (2:2:1, *v*/*v*). Following vortexing and ultrasonic extraction (30 min, 4 °C), the mixture was kept at −20 °C for 10 min. After centrifugation at 14,000× *g* (20 min, 4 °C), the supernatant was collected and evaporated to dryness under vacuum. For final analysis, the residue was redissolved in 100 μL of 50% acetonitrile, vortexed, and centrifuged at 14,000× *g* for 15 min at 4 °C to remove any remaining particulates. The final supernatant was collected for injection. Quality Control (QC) samples were prepared by pooling equal aliquots from each test sample to equilibrate the LC-MS system, monitor instrument performance, and ensure experimental reproducibility.

Metabolite separation was performed using an Agilent 1290 Infinity UHPLC (Agilent Technologies, Santa Clara, CA, USA) system equipped with a HILIC column. The column temperature was maintained at 25 °C, with a flow rate of 0.5 mL/min and an injection volume of 2 μL. Mobile phase A consisted of water containing 25 mM ammonium acetate and 25 mM ammonium hydroxide, while mobile phase B was 100% acetonitrile. The gradient elution program was optimized as follows: 0–0.5 min, 95% B; 0.5–7 min, 95–65% B; 7–8 min, 65–40% B; 8–9 min, 40% B; 9–9.1 min, 40–95% B; and 9.1–12 min, 95% B. Samples were kept in a 4 °C autosampler throughout the analysis, with QC samples inserted into the sequence at regular intervals.

Mass spectrometry was conducted using an AB Triple TOF 6600 system to acquire both primary (MS1) and secondary (MS2) spectra. (AB Sciex Pte. Ltd., Singapore) Electrospray ionization (ESI) source parameters were set as follows: Gas1/Gas2, 60 psi; curtain gas (CUR), 30 psi; ion source temperature, 600 °C; and spray voltage, ±5500 V. The MS1 m/z range was 60–1000 Da with an accumulation time of 0.20 s/spectra. The MS2 m/z range was 25–1000 Da with an accumulation time of 0.05 s/spectra. Data-dependent acquisition (DDA) was performed using the Information-Dependent Acquisition (IDA) mode with a declustering potential of ±60 V and a collision energy of 35 ± 15 eV. Isotope ions were dynamically excluded within 4 Da, and 10 fragment spectra were collected per scan.

To facilitate subsequent analysis, raw spectral data were first converted to the MzXML standard using ProteoWizard (version 3.0, build 25292). Then the XCMS algorithm was utilized to perform automated peak alignment and retention time adjustment, effectively normalizing the extracted peak areas across all samples.

### 2.6. Statistical Analysis

For alpha diversity indices, Welch’s *t*-test was used to evaluate differences between groups. Principal Coordinate Analysis (PCoA) based on Bray–Curtis distances was performed to visualize microbial community shifts, with significance assessed via PERMANOVA (*p* < 0.05). To identify specific microbial biomarkers, pairwise intergroup comparisons were conducted using the Wilcoxon rank-sum test. Linear Discriminant Analysis (LDA) Effect Size (LEfSe) was applied to estimate the effect size of each differentially abundant taxon, with an LDA score threshold of >2.0 and *p* < 0.05. Functional metagenomes were predicted based on the OTU abundance table using PICRUSt2 (version 2.6.3) software to infer Kyoto Encyclopedia of Genes and Genomes (KEGG) pathway enrichment.

Metabolite annotation was performed by matching accurate mass and MS/MS fragments against public databases, including MassBank, METLIN, and MoNA, supplemented by an in-house secondary mass spectrometry database. Differentially abundant metabolites (DAMs) were identified based on the following criteria: Variable Importance in Projection (VIP) ≥ 1 from the OPLS-DA model and *p* < 0.05 from Student’s *t*-test, and |log_2_FC| > 1 (fold change >2 or <0.5).

Metabolic pathway enrichment analysis was conducted via the KEGG database, with significance determined by a *p*-value or false discovery rate (FDR) < 0.05 following −log10 transformation. To integrate the two datasets, an O2PLS-DA model was utilized to identify common variation dimensions between the microbiome and metabolome. Spearman correlation analysis and statistical visualization were subsequently performed using the pheatmap package in R the R package pheatmap (version 1.0.12) (Kolde, 2019). To elucidate associations between differential microbial genera and metabolites.

## 3. Results

### 3.1. Analysis of 16S rRNA Gene Sequencing Results

**Figure 1:**
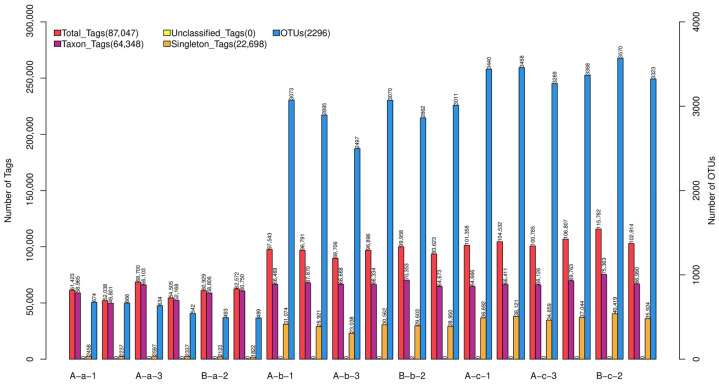
Distribution of sequencing tag counts and OTUs across all samples.

To investigate the effects of NAP supplementation on the gastrointestinal microbiota of Tibetan sheep, 16S rRNA gene sequencing was performed on gastrointestinal samples collected from Groups A and B after 30 days of feeding. After quality control and filtering, more than 80% of raw reads were retained as high-quality sequences for each sample. The number of effective tags exceeded 40,000 per sample, indicating sufficient sequencing depth for reliable downstream analyses. Statistical evaluation of Operational Taxonomic Unit counts (OTUs) and sequencing depth metrics was conducted (Figure 1). Variations in OTUs and tag counts were observed among samples, reflecting differences in microbial richness across groups. These results established the basis for subsequent analyses of microbial community structure and diversity.

### 3.2. Alpha Diversity Analysis

**Figure 2:**
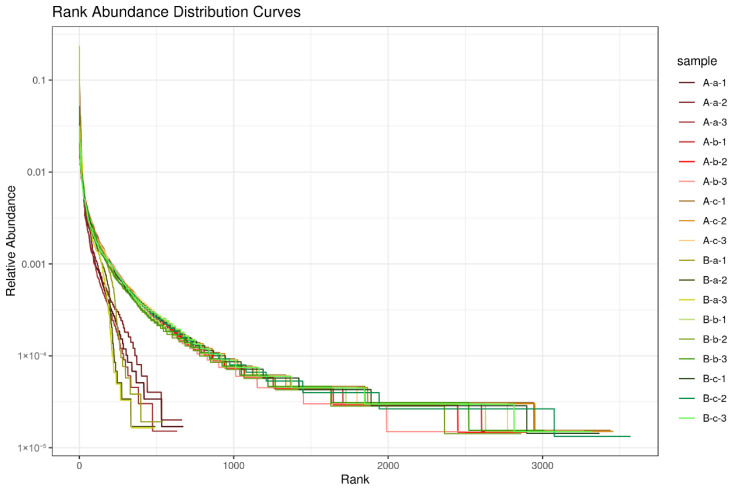
Rank–abundance curves of microbial communities in different samples.

The OTUs in each sample were ranked according to their relative abundance to construct rank–abundance curves. Along the horizontal axis, the length of the curve reflects species richness, with a longer curve indicating a greater number of OTUs. Along the vertical axis, the slope of the curve represents species evenness; a flatter curve indicates a more even distribution of species abundance within the sample. As shown in Figure 2, samples A-a and B-a demonstrated relatively steeper slopes compared with other groups, indicating reduced species evenness. In contrast, the remaining groups exhibited flatter curves, suggesting a more homogeneous distribution of microbial taxa.

Based on Welch’s *t*-test, alpha diversity analysis using the Sob, Chao1, and ACE indices (Table 1) revealed significant differences (*p* < 0.05) in community richness between groups A-a and B-a. However, no significant differences in species richness were observed between groups A-b and B-b or between groups A-c and B-c.

### 3.3. Beta Diversity Analysis

**Figure 3:**
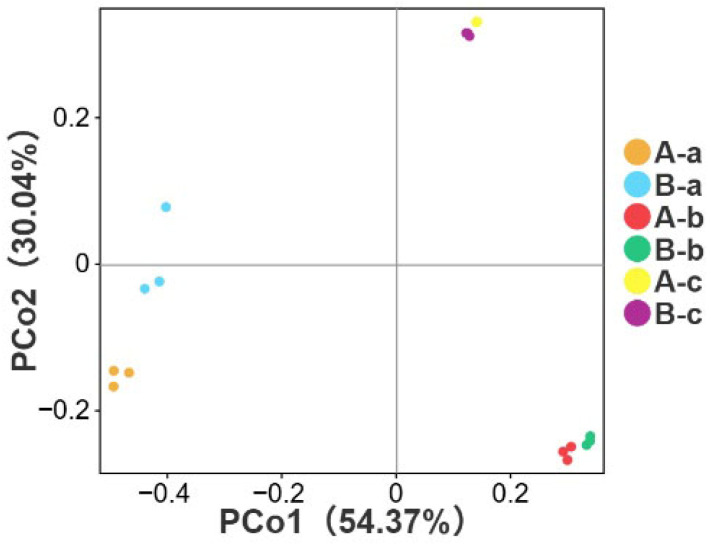
Principal coordinate analysis (PCoA) of microbial community structure among different groups.

PCoA was performed to evaluate differences in microbial community structure among groups. In the PCoA plot, samples positioned closer together indicate more similar microbial community compositions, whereas greater separation reflects increased compositional dissimilarity. As shown in Figure 3, Table 2 and Table 3, a significant difference in microbial community structure was observed between groups A-a and B-a (*p* < 0.05). In contrast, no significant differences were detected between groups A-b and B-b or between groups A-c and B-c (*p* > 0.05). These results were consistent with the alpha diversity analysis. This discrepancy is due to the different analytical focuses of the two statistical methods. The *t*-test evaluates intergroup differences in individual microbial indicators, while PERMANOVA assesses differences in the overall microbial community structure. In this study, NAP affected only certain microbial indicators and did not cause substantial differentiation in the overall community structure, which explains the observed results.

### 3.4. Differential Microbial Composition Analysis

Based on the results of the alpha and beta diversity analyses, significant differences in microbial richness and community structure were observed between groups A-a and B-a, whereas no significant differences were detected between groups A-b and B-b or between groups A-c and B-c. Therefore, subsequent microbial composition analysis focused on groups A-a and B-a. The results are presented in Table 4. The top 10 dominant genera in both groups showed a largely similar taxonomic composition, with *Candidatus_Saccharimonas*, *Christensenellaceae_R7_group*, *Ruminococcus*, *NK4A214_group*, and *Saccharofermentans* constituting the core microbiota. However, notable differences were observed in their relative abundances. In group A-a, *Candidatus_Saccharimonas* was the most abundant genus, whereas in group B-a, *Saccharofermentans* became the predominant genus, and *Candidatus_Saccharimonas* decreased to second place. These findings suggest that NAP supplementation did not alter the core taxonomic composition of the intestinal microbiota in Tibetan sheep but significantly reshaped the relative abundance structure of dominant genera.

**Figure 4:**
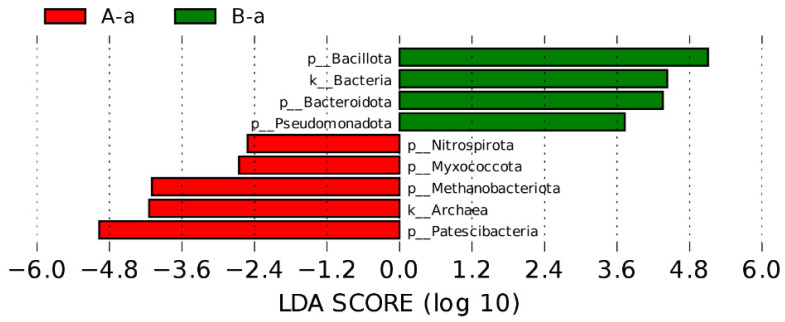
LEfSe analysis identifying differentially enriched taxa between groups A-a and B-a.

Based on the significant differences observed in alpha and beta diversity between groups A-a and B-a, linear discriminant analysis effect size (LEfSe) was performed to identify taxa that were differentially enriched between the two groups. The results are shown in Figure 4. The LEfSe analysis revealed that *Bacillota*, *Bacteria*, *Bacteroidota*, and *Pseudomonadota* were significantly enriched in group B-a. In contrast, *Patescibacteria*, *Archaea*, *Methanobacteriota*, *Myxococcota*, and *Nitrospirota* were significantly enriched in group A-a.

### 3.5. Functional Prediction of Microbial Communities

**Figure 5:**
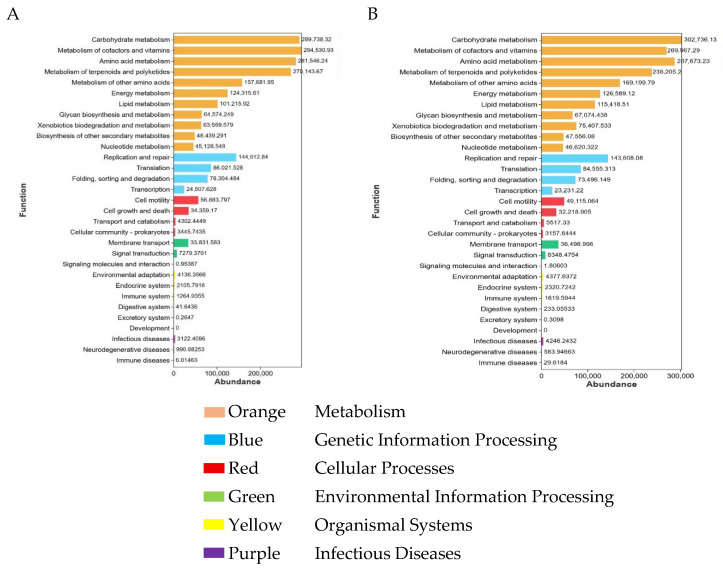
Functional prediction of microbial communities based on KEGG pathways in groups A-a and B-a. Note: (**A**) A-a; (**B**) B-a.

Based on the significant differences observed in alpha diversity, beta diversity, and LEfSe analyses, functional prediction of the microbial communities was conducted. The results are shown in Figure 5. At KEGG, metabolism was the predominant functional category in both groups A-a (Figure 5A) and B-a (Figure 5B). Within this category, carbohydrate metabolism showed the highest relative abundance, followed by metabolism of cofactors and vitamins and amino acid metabolism. Genetic information processing represented the second most abundant functional module in both groups. Although the overall functional profiles were similar between A-a and B-a, differences were observed in the relative abundances of several metabolic pathways, suggesting that NAP supplementation may have modulated functional potential without fundamentally altering core metabolic functions.

The above results indicate that although the overall taxonomic composition of the core microbiota remained stable between groups A-a and B-a, the relative abundances of dominant taxa were altered. This suggests that NAP peptide supplementation may influence gastrointestinal metabolic potential by modulating the proportional distribution of key microbial taxa. Functional prediction analysis further demonstrated that no significant differences were observed in the overall functional gene profiles between the two groups. However, variations in the relative abundances of specific metabolic pathways were detected, implying potential shifts in microbial functional capacity. To further elucidate the impact of NAP on gastrointestinal metabolism in Tibetan sheep, non-targeted metabolomic analysis was subsequently performed. The results are presented below.

### 3.6. Analysis of Non-Targeted Metabolomics Results

**Figure 6:**
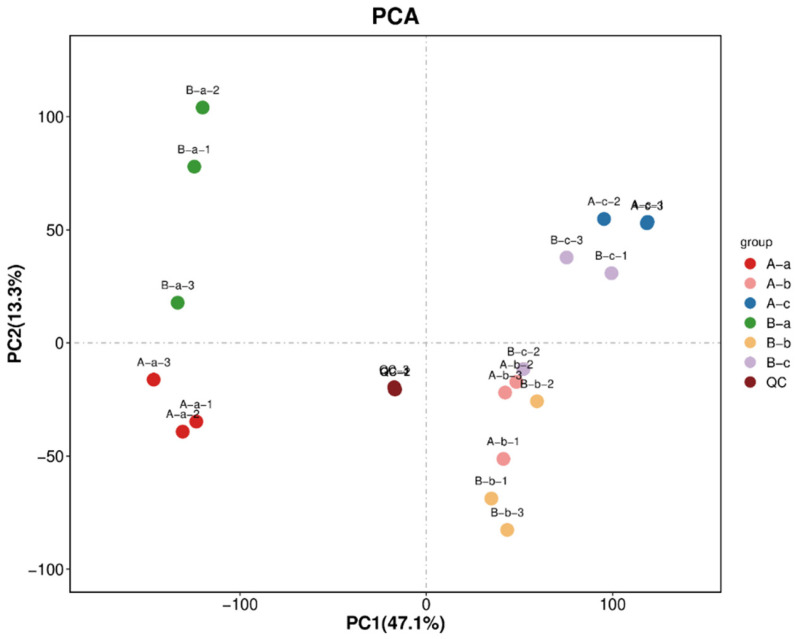
PCA score plot of combined positive and negative ion datasets, including QC samples. Note: A-a-1, A-a-2, and A-a-3 represent three biological replicates of small intestinal contents from control group A-a. A-b-1, A-b-2, and A-b-3 represent three biological replicates of ruminal contents from control group A-b. A-c-1, A-c-2, and A-c-3 represent three biological replicates of rectal contents from control group A-c. B-a-1, B-a-2, and B-a-3 represent three biological replicates of small intestinal contents from experimental group B-a. B-b-1, B-b-2, and B-b-3 represent three biological replicates of ruminal contents from experimental group B-b. B-c-1, B-c-2, and B-c-3 represent three biological replicates of rectal contents from experimental group B-c. QC, quality control; three biological replicates.

To evaluate data quality and analytical stability, the combined positive and negative ion datasets were subjected to principal component analysis (PCA), including QC samples (Figure 6). The PCA score plot showed that QC samples were tightly clustered, with overlapping distribution in the multivariate space, indicating good analytical reproducibility and system stability throughout the experiment. Metabolite identification and annotation were subsequently performed (Table 5). A total of 18,102 metabolite features were detected, including 9793 in positive ion mode and 8309 in negative ion mode. Among these, 3916 positive ion features and 3521 negative ion features were successfully annotated to known metabolites.

### 3.7. Differential Metabolite Profiling Between Groups

**Figure 7:**
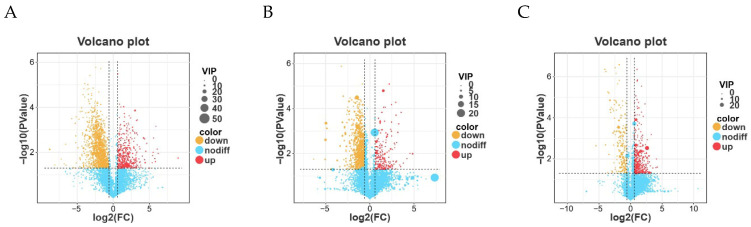
Volcano plots showing differential metabolites between A-a and B-a (**A**), A-b and B-b (**B**), and A-c and B-c (**C**).

Based on the abundance matrix containing 7437 annotated metabolite features retained across all samples, differential metabolite analysis was performed between Group A (control) and Group B (experimental). Metabolites were screened using the criteria of log_2_ fold change (FC > 1) or (FC < −1) and *p* < 0.05. The distribution of differential metabolites is presented as volcano plots in Figure 7. In the plots, red dots represent upregulated metabolites, whereas yellow dots indicate downregulated metabolites. Overall, more metabolites were downregulated than upregulated in all comparisons. Notably, several downregulated metabolites exhibited high variable importance in projection (VIP) values, suggesting that these metabolites contributed substantially to group discrimination and may represent key metabolites distinguishing Group A from Group B.

**Figure 8:**
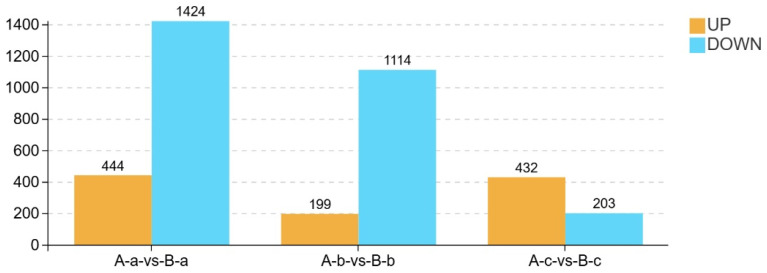
Summary of differentially expressed metabolites in combined ion mode.

A statistical summary of differentially expressed metabolites between groups is presented in Figure 8. In the comparison between A-a and B-a, 444 metabolites were upregulated and 1424 were downregulated in group B-a relative to group A-a. Similarly, in the A-b vs. B-b comparison, 199 metabolites were upregulated and 1114 were downregulated. In contrast, the A-c vs. B-c comparison showed 432 upregulated and 203 downregulated metabolites. Overall, the number of downregulated metabolites exceeded that of upregulated metabolites in the A-a vs. B-a and A-b vs. B-b comparisons, whereas the opposite trend was observed in the A-c vs. B-c comparison.

**Figure 9:**
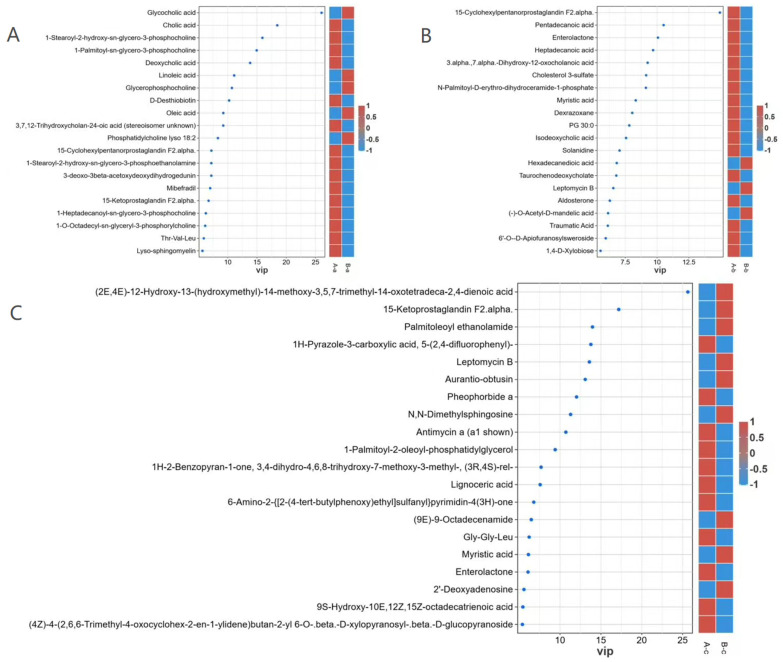
VIP plots of differential metabolites for A-a vs. B-a (**A**), A-b vs. B-b (**B**), and A-c vs. B-c (**C**).

Differential metabolite analysis further identified key discriminative metabolites between groups A and B based on variable importance in projection (VIP) scores derived from the OPLS-DA model (Figure 9). In the A-a vs. B-a comparison, glycocholic acid, linoleic acid, glycerophosphocholine, oleic acid, and lysophosphatidylcholine (18:2) were enriched in group B-a, whereas cholic acid, 1-stearoyl-2-hydroxy-sn-glycero-3-phosphocholine, 1-palmitoyl-sn-glycero-3-phosphocholine, deoxycholic acid, D-desthiobiotin, trihydroxycholan-24-oic acid, and 15-cyclohexylpentanorprostaglandin F2α were enriched in group A-a. In the A-b vs. B-b comparison, hexadecanedioic acid, leptomycin B, and (−)-O-acetyl-D-mandelic acid were enriched in group B-b, whereas 15-cyclohexylpentanorprostaglandin F2α, pentadecanoic acid, enterolactone, heptadecanoic acid, 3α,7α-dihydroxy-12-oxocholanoic acid, and cholesterol sulfate were enriched in group A-b. Similarly, in the A-c vs. B-c comparison, (2E,4E)-12-hydroxy-13-(hydroxymethyl)-14-methoxy-3,5,7-trimethyl-14-oxotetradeca-2,4-dienoic acid, 15-ketoprostaglandin F2α, palmitoleoyl ethanolamide, leptomycin B, and aurantio-obtusin were enriched in group B-c, whereas 1H-pyrazole-3-carboxylic acid, pheophorbide a, antimycin A, and 1-palmitoyl-2-oleoyl-phosphatidylglycerol were enriched in group A-c.

### 3.8. KEGG Pathway Enrichment Analysis of Differential Metabolites Between Groups

**Figure 10:**
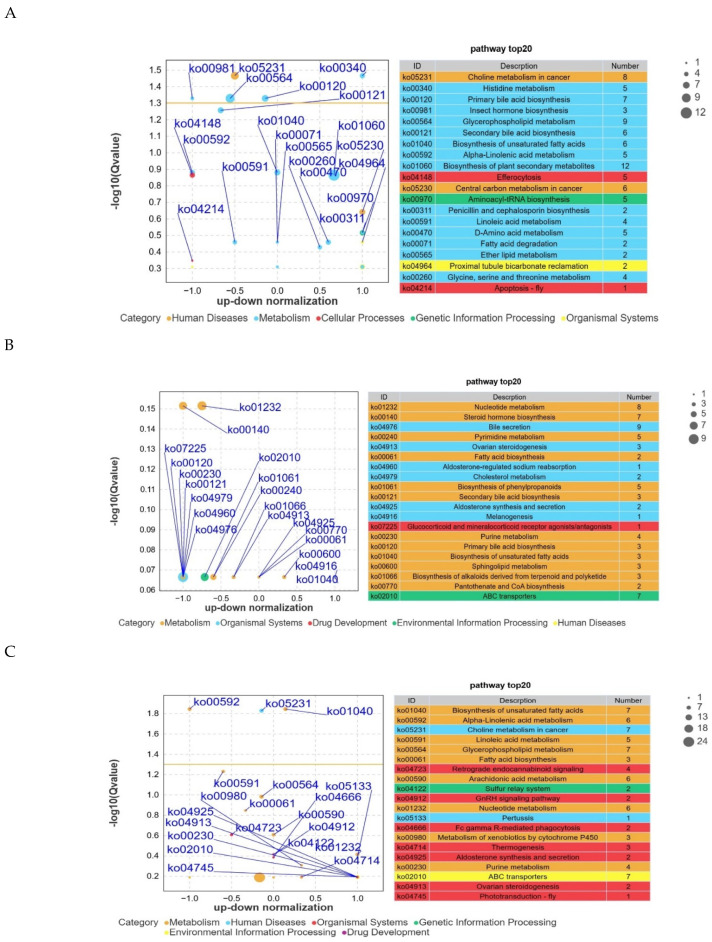
KEGG pathway enrichment analysis of differential metabolites. Note: The yellow line indicates the significance threshold corresponding to *p* = 0.05 (−log10 *p*-value). Pathways above this threshold are considered significantly enriched. (**A**) A-a vs. B-a KEGG pathway enrichment analysis of differential metabolites; (**B**) A-b vs. B-b KEGG pathway enrichment analysis of differential metabolites; (**C**) A-c vs. B-c KEGG pathway enrichment analysis of differential metabolites.

Differential metabolites were mapped to the KEGG database for pathway enrichment analysis. A hypergeometric test was applied to identify pathways significantly enriched in differential metabolites relative to the overall metabolite background. As shown in Figure 10A, several pathways were significantly enriched in the A-a vs. B-a comparison, including choline metabolism in cancer, histidine metabolism, and primary bile acid biosynthesis. In contrast, no significantly enriched pathways were detected in the A-b vs. B-b comparison (Figure 10B). Only a limited number of pathways were enriched in the A-c vs. B-c comparison (Figure 10C), including alpha-linolenic acid metabolism and biosynthesis of unsaturated fatty acids.

Non-targeted metabolomics analysis indicated that, unlike the significant pathway enrichment observed in the A-a vs. B-a comparison, no significantly enriched pathways were detected between A-b and B-b. This suggests that the differential metabolites identified in these two subgroups did not converge into coordinated pathway-level alterations but were more likely to represent isolated metabolic fluctuations with limited systemic impact. In contrast, a small number of pathways were enriched in the A-c vs. B-c comparison, including alpha-linolenic acid metabolism and biosynthesis of unsaturated fatty acids. Combined with the profile of differential metabolites in this subgroup, these findings suggest potential localized modulation of lipid metabolism. However, no extensive pathway activation or inhibition was observed. Overall, these results indicate that the most pronounced and coordinated metabolic alterations occurred in the A-a vs. B-a comparison, highlighting this subgroup as the primary focus of metabolic regulation in the present study.

### 3.9. Integrated Analysis of 16S rRNA Gene Sequencing and Non-Targeted Metabolomics

To further investigate the overall associations between the intestinal microbiota (genus level) and the metabolomic profiles of Tibetan sheep following NAP supplementation, an integrative multi-omics analysis was conducted. An orthogonal two-way partial least squares (O2PLS) model was constructed to evaluate the covariance structure between the microbiome and metabolome datasets. The contribution statistics of the O2PLS model are summarized in Table 6.

**Figure 11:**
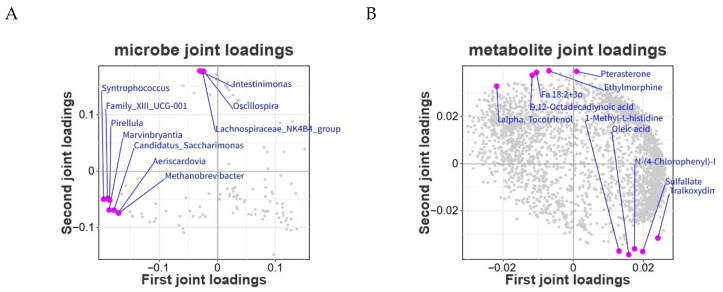
O2PLS loading plot for integrated analysis of genus-level microbiota and metabolomic profiles. Note: (**A**) microbiota; (**B**) metabolomic profiles.

**Figure 12:**
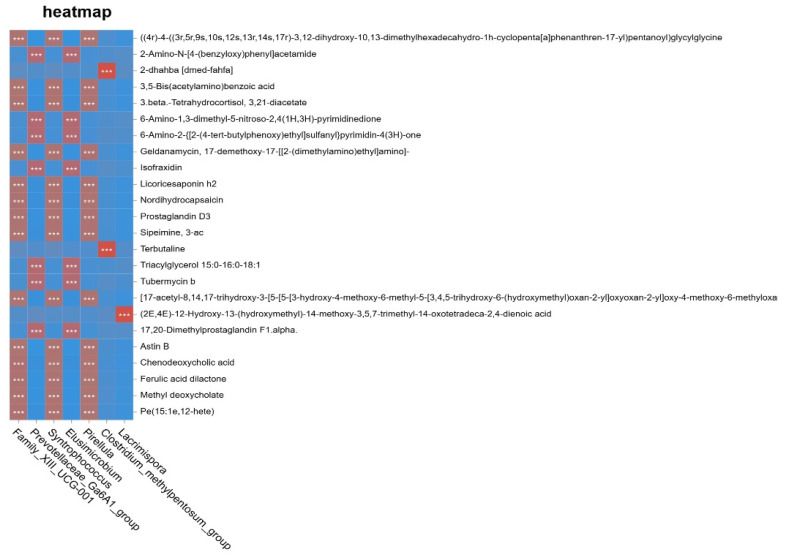
Correlation heatmap of integrated microbiome–metabolome analysis at the genus level. Note: The horizontal axis represents genera, and the vertical axis represents differential metabolites. Each cell indicates the Spearman correlation coefficient between microbial abundance and metabolite levels. Red denotes significant positive correlations, and blue denotes significant negative correlations (*p* < 0.05). Asterisks denote statistical significance: *** represents an extremely high significance level (*p* < 0.001).

The shared variation between the microbiome and metabolome datasets was extracted using the O2PLS model. As shown in Figure 11A, genera such as *Syntrophococcus* and *Family_XIII_UCG-001* were distributed on the left side of the loading plot, whereas *Intestinimonas* and *Oscillospira* were located on the right side, indicating distinct contributions of different genera to the integrative model. Similarly, in Figure 11B, metabolites such as prenosterone and ethylmorphine were positioned in the upper right quadrant, while talkoxydir and sulfate were located in the lower right quadrant, suggesting functional differentiation among metabolites within the integrated association structure. To further clarify the association patterns between the intestinal microbiota (genus level) and metabolomic profiles, a Spearman correlation heatmap was constructed using the R package pheatmap based on genus-level microbial abundance and differential metabolites. The results are shown in Figure 12. Metabolites and genera clustered within the same color blocks exhibited similar correlation patterns, implying potential involvement in related metabolic pathways or physiological processes. Terbutaline and 2-dhahba (dmed-fahfa) showed significant positive correlations (*p* < 0.05) with certain genera, including *Lacrimispora*. In contrast, most metabolites, such as prostaglandin D3 and chenodeoxycholic acid, displayed significant negative correlations (*p* < 0.05) with genera including *Lacrimispora* and *Clostridium_methylpentosum*.

**Figure 13:**
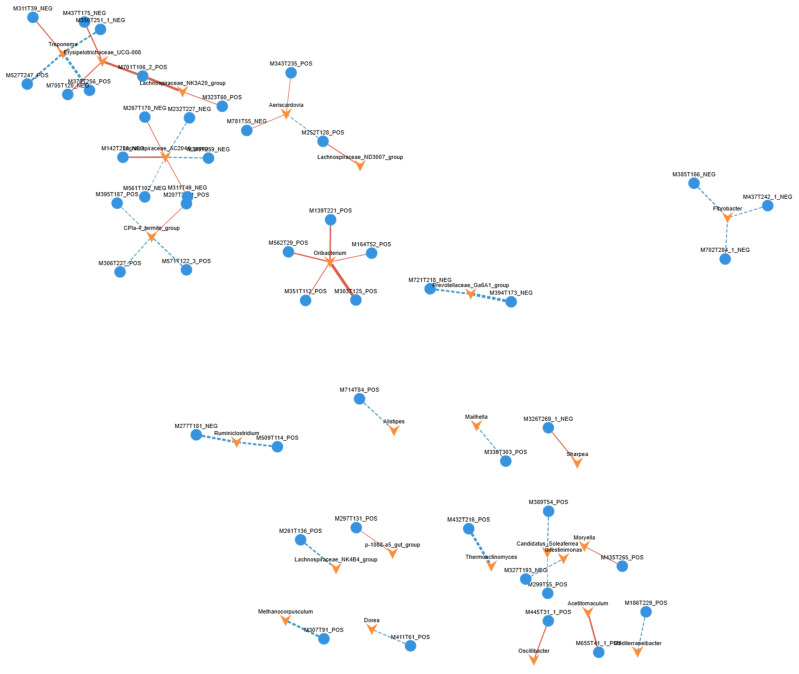
Integrated microbiome–metabolome correlation network at the genus level. Note: Blue nodes represent metabolites, and orange nodes represent microbial genera. Red edges indicate significant positive correlations, whereas blue edges indicate significant negative correlations. Edge thickness reflects the strength of the correlation coefficient, and node size corresponds to the degree of connectivity (number of associations).

The correlation network exhibited a clear modular structure (Figure 13), in which distinct microbial genera and metabolites formed relatively independent interaction clusters, reflecting the complexity and specificity of microbiome–metabolome associations in the intestinal ecosystem of Tibetan sheep. Within the network, genera such as *Lachnospiraceae_NK4A136_group*, *Dialister*, and *Aerotrigona* functioned as highly connected nodes, suggesting their potential roles as key hubs in host metabolic regulation. *Lachnospiraceae_NK4A136_group* is known to participate in short-chain fatty acid (SCFA) production, and its positive correlations with specific metabolites may indicate its involvement in dietary fiber fermentation and subsequent metabolic processes. *Dialister*, an acid-producing genus, showed associations with metabolites potentially related to intestinal acidification and barrier function. Similarly, the positive correlations observed between *Aerotrigona* and several metabolites may reflect its potential contribution to carbohydrate metabolism or host energy regulation.

## 4. Discussion

Quality control results demonstrated adequate sequencing depth and a high proportion of high-quality reads, meeting the technical specifications for gastrointestinal microbial research in ruminants [28], which indicates that the dataset was robust and reliable for downstream analyses. Rank–abundance and alpha diversity analyses revealed significant differences in microbial richness and evenness in the small intestinal contents between the experimental and control groups. Consistently, beta-diversity analysis also showed a clear separation between the small intestinal microbial communities of the two groups, whereas no significant differences were observed at the other gastrointestinal sites. These findings suggest that NAP supplementation exerts site-specific effects on the intestinal microbiota of Tibetan sheep, with the small intestine appearing particularly responsive. Although the dominant core genera were largely conserved between groups, shifts in their relative abundances were observed. Functional prediction analysis further indicated that, while the overall functional gene profiles were not significantly altered, variations in the relative abundance of specific metabolic pathways were detected. For example, the most abundant pathway in one group was metabolism of cofactors and vitamins, whereas carbohydrate metabolism was the most abundant pathway in the other group.

Metabolomic analysis further demonstrated distinct metabolic profiles between the experimental and control groups. The tight clustering of QC samples confirmed the stability and reproducibility of the analytical platform [29]. In Group A, higher levels of cholic acid, deoxycholic acid, and trihydroxycholan-24-oic acid were observed. These bile acids are central components of bile acid metabolism and play essential roles in cholesterol conversion, lipid digestion, and nutrient absorption. Previous studies have also highlighted the close relationship between bile acid homeostasis, intestinal microecology, and lipid metabolism [30]. Additionally, elevated levels of phosphatidylcholine-related metabolites, including 1-stearoyl-2-hydroxy-sn-glycero-3-phosphocholine and 1-palmitoyl-sn-glycero-3-phosphocholine, may reflect the maintenance of membrane structural integrity and lipid transport capacity [31]. The higher abundance of D-desthiobiotin and related biotin derivatives further suggests active participation in fundamental metabolic processes such as fatty acid synthesis and amino acid metabolism. Collectively, these findings indicate that the metabolic profile of Group A was characterized by relatively stable bile acid and phospholipid metabolism. In contrast, Group B exhibited increased levels of unsaturated fatty acids, including linoleic acid and oleic acid. These metabolites are closely associated with lipid metabolic balance, membrane fluidity, and inflammatory regulation [32]. Unsaturated fatty acids not only possess anti-inflammatory properties but may also improve cellular energy metabolism by altering the composition and fluidity of cell membranes, thereby providing protective effects under conditions of metabolic stress [33]. Taken together, the coordinated alterations in bile acid and phospholipid metabolism in Group B suggest a restructuring of lipid metabolic networks rather than a complete disruption of metabolic homeostasis.

Differential metabolites were mapped to the KEGG database for enrichment analysis, and significantly enriched pathways were identified using hypergeometric tests [34]. In the comparison between A-a and B-a, clear functional links were observed between the differential metabolites and the enriched pathways. Group A-a appeared to maintain metabolic homeostasis through higher levels of bile acids and phosphatidylcholines, which were associated with primary bile acid biosynthesis, choline metabolism, and histidine metabolism. By contrast, Group B-a showed altered metabolic flux through these pathways, accompanied by increased levels of conjugated bile acids, phospholipid metabolites, and unsaturated fatty acids. Differential metabolites reflect the most direct physiological changes following NAP intervention and may serve as molecular indicators of treatment effects, whereas pathway enrichment analysis integrates these metabolite changes to reveal broader regulatory networks, biological functions, and potential mechanisms of action.

Regarding microbiome–metabolome coupling, the dynamic interaction between the gut microbial community and host metabolic networks is regarded as a core mechanism for maintaining host physiological homeostasis [35]. Preliminary results showed significant positive correlations between terbutaline, 2-dhahba [dmed-fahfa], and the genus Lacrimispora, suggesting that this genus may contribute to the synthesis, transformation, or accumulation of metabolites related to fatty acid or steroid metabolism. As a fermentative bacterium in the gut belonging to the phylum Firmicutes, Lacrimispora is frequently involved in fatty acid and steroid metabolism, and its abundance may be linked to the regulation of related metabolic pathways [36], thus forming a potential metabolic–microbial coupling circuit. In addition, most metabolites, such as prostaglandin D3 and chenodeoxycholic acid, were significantly negatively correlated with genera including Lacrimispora and Clostridium_methylpentosum. As a key member of the secondary bile acid pool, chenodeoxycholic acid participates in lipid digestion and absorption as well as microecological regulation [37], and its inhibitory effect on Firmicutes has been reported in other studies [38]. This negative correlation is therefore consistent with the literature on bile acid-mediated microecological regulation, indicating that bile acid metabolites may help maintain gut microbial balance by inhibiting the growth of specific bacteria and thereby preventing inflammation and metabolic disturbances caused by local dysbiosis.

Collectively, this study reveals multilevel ecological responses of the Tibetan sheep gut to nano-antimicrobial peptide intervention. The small intestine showed higher sensitivity to exogenous intervention, reflected by the separation of microbial community structure and the dynamic adjustment of related metabolic pathways. At the metabolic level, differential expression of bile acid, fatty acid, and phospholipid metabolic pathways suggests that the intervention may affect host energy balance and membrane structure/function through the coupling of lipid metabolism and bile acid homeostasis [39]. Further microbiome–metabolome coupling analysis revealed that, within the covariation dimension of bile acid metabolism and lipid-related metabolites [40], specific genera (e.g., Lacrimispora) were significantly associated with several metabolites, implying that potential regulatory networks may maintain intestinal ecological balance through microbial production or transformation of metabolites and feedback regulation of microbial habitats by metabolites [41].

It should be emphasized that the above conclusions are based on the current data and should be interpreted with caution. This study used terminal sampling, which reflects only static results at a single time point and cannot reveal the dynamic changes in microorganisms and metabolites over time after intervention. Second, the small sample size may compromise the robustness and generalizability of the statistical results. Finally, the study lacks direct host phenotypic measurements, such as growth performance, intestinal morphology, and inflammatory markers, making it difficult to establish direct links among microbiota, metabolites, and host phenotypes. These limitations will be addressed in future studies. In addition, nano-antimicrobial peptide (NAP) was supplemented only in the treatment group in this study; therefore, the potential influence of trace exogenous protein supplementation on the experimental results cannot be completely excluded. Ideally, scrambled peptides with identical amino acid compositions but disrupted sequences should be included as controls to distinguish the specific biological activity of NAP from the nonspecific effects of additional protein supplementation. Due to practical limitations in the present study, such a control was not included in the experimental design. However, the supplemental dose of NAP used in this study was very low (125 mg/day), contributing less than 0.02% of the total dietary protein intake, and was therefore considered unlikely to substantially affect the overall nutritional intake of the animals. Therefore, we speculate that the observed changes in microbiota and production performance were primarily associated with the antibacterial and regulatory activities of NAP rather than with simple protein supplementation. Future studies incorporating scrambled peptide controls will be conducted to further verify the mechanism of action of NAP.

## 5. Conclusions

This study demonstrates that the small intestine is the primary site of microecological response to NAP supplementation in Tibetan sheep. While the core microbiota remained relatively stable, NAP significantly reshaped the microbial community structure and diversity (*p* < 0.05). Integrated omics analysis revealed that these microbial shifts were associated with systematic alterations in bile acid, fatty acid, and phospholipid metabolic pathways. Specifically, the strong correlations between key genera and metabolites, such as bile acids and prostaglandins, suggest that NAP may contribute to the maintenance of intestinal homeostasis by regulating intestinal microecology and host metabolism.

## Figures and Tables

**Table 1:** Alpha diversity comparison based on the Sob, Chao1, and ACE indices.

Group	Sob (*p*-Value)	Chao1 (*p*-Value)	ACE (*p*-Value)
A-a vs. B-a	0.0029	0.0191	0.0062
A-b vs. B-b	0.4549	0.4242	0.4097
A-c- vs. B-c	0.7635	0.7670	0.8353

**Table 2:** Welch’s *t*-test analysis of beta diversity among groups.

Group	*p*-Value
A-a vs. B-a	0.03
A-b vs. B-b	0.14
A-c vs. B-c	0.07

**Table 3:** Results of the Beta diversity analysis using PERMANOVA.

Group	Df	Sums of Sqs	Mean Sqs	F-Value	R^2^	*p*-Value
A-a vs. B-a	1	0.7326	0.7326	7.4949	0.652	0.1
A-b vs. B-b	1	0.5624	0.5624	21.9001	0.8456	0.1
A-c vs. B-c	1	0.7028	0.7028	31.5081	0.8873	0.1

**Table 4:** Relative abundances (%) of the top 10 dominant genera in groups A-a and B-a (mean ± SD).

Genus	A-a-1/B-a-1	A-a-2/B-a-2	A-a-3/B-a-3	Mean ± SD
Group A				
Candidatus_Saccharimonas	35.3091	28.1601	36.7487	33.4060 ± 4.5997
Christensenellaceae_R-7_group	3.0001	3.7911	3.0468	3.2793 ± 0.4438
Ruminococcus	2.7321	2.8092	1.8880	2.4764 ± 0.5111
NK4A214_group	1.5246	1.5743	2.1102	1.7364 ± 0.3247
Saccharofermentans	1.3109	1.0843	1.6868	1.3607 ± 0.3043
Xylanibacter	0.2273	0.9036	0.2723	0.4677 ± 0.3781
UCG-005	0.0594	0.0161	0.0545	0.0433 ± 0.0237
Rikenellaceae_RC9_gut_group	0.0356	0.4377	0.0257	0.1663 ± 0.2351
Treponema	0.0153	0.0281	0.0076	0.0170 ± 0.0104
Bacteroides	0.0034	0.0080	0.0045	0.0053 ± 0.0024
Group B				
Saccharofermentans	12.7933	20.9417	23.6148	19.1166 ± 5.6369
Candidatus_Saccharimonas	16.1843	11.4155	9.0831	12.2276 ± 3.6196
Christensenellaceae_R-7_group	3.1475	4.6747	5.0815	4.3012 ± 1.0197
NK4A214_group	3.0114	2.2583	2.5926	2.6208 ± 0.3774
Xylanibacter	3.0133	0.5459	0.2699	1.2764 ± 1.5105
Ruminococcus	1.6600	0.94038	1.0140	1.2048 ± 0.3959
Treponema	0.9929	0.0002	0.0082	0.3338 ± 0.5708
Rikenellaceae_RC9_gut_group	0.7150	0.0238	0.0280	0.2556 ± 0.3979
UCG-005	0.1591	0.0357	0.0807	0.0918 ± 0.0624
Bacteroides	0.0077	0.0017	0.0066	0.0053 ± 0.003

**Table 5:** Summary of metabolite detection and annotation results in positive and negative ion modes.

Type	All	Known	Unknown
POS	9793	3916	5877
NEG	8309	3521	4788

**Table 6:** O2PLS model performance and contribution statistics for integrated microbiome–metabolome analysis.

Model	R2X	R2Y	R2Xcorr	R2Ycorr
Genus	0.838	0.873	0.664	0.838

## Data Availability

The raw sequencing data (16S rRNA gene) have been deposited in the NCBI Sequence Read Archive (SRA: PRJNA1451483), and the non-targeted metabolomics data have been submitted to the MetaboLights database. Accession numbers will be provided upon the formal acceptance of the manuscript. For further inquiries regarding the datasets, please contact the corresponding author.
